# Octacalcium phosphate crystals directly stimulate expression of inducible nitric oxide synthase through p38 and JNK mitogen-activated protein kinases in articular chondrocytes

**DOI:** 10.1186/ar1763

**Published:** 2005-05-27

**Authors:** Hang-Korng Ea, Benjamin Uzan, Christian Rey, Frédéric Lioté

**Affiliations:** 1 INSERM U606, Centre Viggo Petersen, Hôpital Lariboisière, Paris, France; 2 CIRIMAT, UMR CNRS 5085, ENSIACET, Toulouse, France; 3 Université Paris 7, UFR Saint-Louis Lariboisière, Paris, France

## Abstract

Basic calcium phosphate (BCP) crystals, including hydroxyapatite, octacalcium phosphate (OCP) and carbonate-apatite, have been associated with severe osteoarthritis and several degenerative arthropathies. Most studies have considered the chondrocyte to be a bystander in the pathogenesis of calcium crystal deposition disease, assuming that synovial cell cytokines were the only triggers of chondrocyte activation. In the present study we identified direct activation of articular chondrocytes by OCP crystals, which are the BCP crystals with the greatest potential for inducing inflammation. OCP crystals induced nitric oxide (NO) production and inducible nitric oxide synthase (NOS) mRNA expression by isolated articular chondrocytes and cartilage fragments, in a dose-dependent manner and with variations over time. OCP crystals also induced IL-1β mRNA expression. Using pharmacological and cytokine inhibitors, we observed that OCP crystals induced NO production and inducible NOS mRNA activation were regulated at both the transcriptional and the translational levels; were independent from IL-1β gene activation; and involved p38 and c-Jun amino-terminal kinase (JNK) mitogen-activated protein kinase (MAPK) pathways, as further confirmed by OCP crystal-induced p38 and JNK MAPK phosphorylation. Taken together, our data suggest that the transcriptional inducible NOS response to OCP crystals involved both the p38 and the JNK MAPK pathways, probably under the control of activator protein-1. NO, a major mediator of cartilage degradation, can be directly produced by BCP crystals in chondrocytes. Together with synovial activation, this direct mechanism may be important in the pathogenesis of destructive arthropathies triggered by microcrystals.

## Introduction

Crystals of calcium pyrophosphate dihydrate (CPPD) and basic calcium phosphate (BCP), including octacalcium phosphate (OCP), carbonate-substituted apatite and tricalcium phosphate, are the calcium-containing crystals most commonly associated with articular and periarticular disorders. BCP crystals can cause acute attacks of inflammatory arthritis [1] or acute calcific periarthritis [2], and in a few patients they result in erosive arthritis [3]. More often, they are associated with an exaggerated form of osteoarthritis (OA) or with joint destruction [4-7]. The prevalence of CPPD and BCP microcrystals in patients with joint disease increases significantly with ageing. These microcrystals have been identified in 60% of joint fluids from patients with knee OA undergoing total arthroplasty [7,8]. More specifically, the presence of BCP crystals correlates strongly with radiographic evidence of cartilaginous degeneration [7,8]. Physical interactions between chondrocytes and BCP crystals could occur *in vivo *in various settings. BCP crystals can be released from subchondral bone through cartilage lesions. Interestingly, hypertrophic chondrocytes, which are present in the superficial zone of osteoarthritic cartilage, can produce calcifying apoptotic bodies, resulting in BCP formation in the perichondrocytic milieu [9].

The mechanism of cartilage degradation in BCP crystal-associated OA remains unclear. Hypotheses include synovial lining cell stimulation by BCP crystals, resulting in synovial cell proliferation [10-12], release of matrix-degrading molecules [13-20], and secretion of inflammatory mediators [21] and cytokines that, in turn, stimulate chondrocytes to generate matrix-degrading molecules [12,13,16,22,23].

Most studies have considered chondrocytes as passive bystanders in the pathogenesis of BCP crystal associated OA and CPPD disease. However, in primary OA chondrocytes appear to play a major role in cartilage damage. In immunohistochemistry studies chondrocytes expressed larger amounts of inflammatory mediators and cytokines, such as IL-1β and tumour necrosis factor (TNF)-α than did OA synoviocytes [24], suggesting an active role for chondrocytes in cartilage destruction. *In vitro*, BCP crystals induced prostaglandin secretion [13], collagenase [12] and metalloproteinase (MMP)-13 mRNA accumulation, and MMP-13 protein secretion by articular chondrocytes [16].

Osteoarthritic lesions may result from an imbalance between anabolic and catabolic processes. Nitric oxide (NO) is a pleiotropic mediator that is intimately involved in the OA catabolic process [25-28]. NO is synthesized via L-arginine oxidation by a family of nitric oxide synthases (NOSs). Of the three known NOS isomers, two are constitutively expressed (neural ncNOS or NOS-1 and endothelium ecNOS or NOS-3) and one is inducible (iNOS or NOS-2). Expression of iNOS has been demonstrated in various cell types. Within the joint, chondrocytes may be the main cell source of NO, and iNOS expression is increased in human OA cartilage [29]. In animal models of OA, treatment with the specific iNOS inhibitor *N*-iminoethyl-L-lysine significantly reduced the progression of structural changes [30,31]. This structural effect was accompanied by reductions in MMP synthesis, IL-1β and prostaglandin E_2 _production, and chondrocyte apoptosis [32].

BCP crystals are heterogeneous in terms of their ultrastructure and physicochemical composition, and previous studies [33,34] have shown differences in their phlogistic properties. We studied OCP crystals, which are the BCP crystals that produced the greatest degrees of inflammation in earlier studies [33,34]. Although OCP is one of the BCP crystals found in joints [35], its biological significance is unclear. It could be deleterious to cartilage at some stages, resulting in inflammatory reaction, but it could be also a precursor to hydroxyapatite, which is a BCP crystal found at greater concentration in joints but with reduced inflammatory capability. We postulated a direct effect of BCP crystals on chondrocyte activation.

To investigate this hypothesis, we looked for effects of OCP crystals on NO production by bovine cartilage organ cultures and isolated articular chondrocytes. We also examined whether OCP crystals activated iNOS expression through the protein kinase signal transduction pathway involving Erk1/2 (p42/44), p38, and c-Jun amino-terminal kinase (JNK) mitogen-activated protein kinases (MAPKs). Finally, we investigated whether IL-1β release triggered by OCP crystals acted as a secondary messenger of OCP crystal induced iNOS expression.

## Materials and methods

### Reagents

Foetal bovine serum (FBS) was obtained from Dominique Dutscher (Brumath, France). Dulbecco's modified Eagle's medium (DMEM) with high glucose (4.5%), phosphate-buffered saline, penicillin, streptomycin, fungizone, Taq polymerase, M-MLV reverse transcriptase, dNTP set, primers and TRIzol reagents were obtained from Invitrogen (Cergy-Pontoise, France). The pharmacological MAPK inhibitors PD98059 and SB203580, and JNK II inhibitor were purchased from Calbiochem (San Diego, CA, USA). *N*^G^-nitro-L-arginine methyl ester (L-NAME), cycloheximide, actinomycin D, pepstatin, aprotinin, leupeptin, phenylmethyl sulfofluoride, poly-(2-hydroxyethyl methacrylate; poly-HEMA) and bacterial collagenase type II were obtained from Sigma-Aldrich (St Quentin Fallavier, France). IL-1β and IL-1 receptor antagonist (IL-1ra) were purchased from R&D systems Inc. (Abingdon, Oxford, UK).

### Antibodies

Phospho-specific JNK (Thr183/Tyr185) and p38 (Thr180/Tyr182), and total JNK and p38 antibodies were purchased from Cell Signaling Technology (Ozyme, St Quentin Yvelines, France). Polyclonal horseradish peroxidase-conjugated goat anti-rabbit IgG was obtained from Sigma-Aldrich.

### Articular cartilage organ cultures

Carpal–metacarpal joints of calves (<3 years of age) were provided by a local French slaughterhouse. Cartilage disks (15–25 mg) were aseptically dissected from articular cartilage slices and washed three times in DMEM containing 100 μg/ml streptomycin, 100 IU/ml penicillin, and 0.25 μg/ml fungizone. Disks were transferred to 96-well, flat-bottomed plates (TPP; ATGC biotechnologie, Marne la Vallée, France) containing DMEM with high glucose supplemented with 10% heat-inactivated FBS and antibiotics (hereafter referred to as 'complete medium') and cultured at 37°C in a humidified atmosphere supplemented with 5% carbon dioxide. The medium was changed 72 hours later to 200 μl DMEM with 1% FBS, and OCP crystals or recombinant human IL-1β were added 24 hours later.

### Chondrocyte isolation and culture

Bovine chondrocytes were isolated from carpal–metacarpal cartilage, as described by Kuettner and coworkers [36]. Briefly, articular cartilage was cut into small pieces, and chondrocytes were released by collagenase digestion using bacterial collagenase type II (0.2% in DMEM) for 20 hours at 37°C with gentle shaking. Chondrocytes were then collected through a 100 μm nylon cell strainer (Cell strainer, Falcon, VWR International, Fontenay sous Bois, France), washed, and plated at high density (10^7^cells/ml) in complete medium. At subconfluence, cells were starved in DMEM with 1% FBS for 24 hours and then harvested and replated at 10^6 ^cells/ml in 96-well, round-bottomed (for nitrite production studies) or 24-well (for MAPK or iNOS activation studies) plates coated with 10% poly-HEMA [37]. Poly-HEMA coating prevents cell adhesion and preserves the articular cartilage phenotype for up to several weeks [38].

### Octacalcium phosphate crystal preparation

Sterile, pyrogen-free OCP crystals (homogeneity in size 1.5 ± 0.5 μm; Ca/[P+CO_3_] ratio 1.33) were synthesized, as described previously [33,39], by suspending calcium hydrogen phosphate dihydrate (5 g) in 300 ml of an aqueous solution of diammonium hydrogen phosphate (5 g) at 37°C for 48 hours. OCP crystal size and morphology were determined using a Phillips EM 300 transmission electron microscope (Philips, Eindhoven, The Netherlands) and their nature by X-ray diffraction (INEL CPS 120 diffractometer; Enraf Nonius SA, Sevran, France) and infrared spectroscopy (Perkin-Elmer FTIR 1760 spectrometer; Courtaboeuf, France) before and after sterilization. Sterilization was by exposure to ^60^Co γ-radiation by the CisBio International Company (Laboratoire des Produits d'Irradiation at the Commissariat à l'Energie Atomique, Saclay, France). OCP crystals were confirmed to be pyrogen-free, as shown previously [33].

### Determination of nitrite level

NO accumulation was measured by the Griess reaction. Confluent articular chondrocytes were seeded at a concentration of 10^6 ^cells/ml as described above. Chondrocytes were treated with various pharmacological inhibitors for 60 min, and then stimulated by various doses of OCP crystals or IL-1β. For the indicated times in the cell cultures and 4 days later in the cartilage organ culture, 50 μl cell-free supernatants from chondrocyte or articular cartilage organ cultures were mixed with 150 μl Griess reagent. The NO_2_^- ^concentrations were immediately determined by measuring absorbance at 550 nm in an enzyme-linked immunosorbent assay plate reader. NO measurements are expressed as μmol/l in culture supernatants and as μmol/l per mg cartilage where appropriate.

### Preparation of cytoplasmic extracts for mitogen-activated protein kinase studies

Nonadherent chondrocytes were treated with various pharmacological inhibitors for 60 min then stimulated with OCP crystals or IL-1β. For the indicated times, chondrocytes were collected and placed in lysis buffer (20 mmol/l Tris.HCl [pH 7.5], 150 mmol/l NaCl, 1% Triton X-100, 1 mmol/l EDTA, 1 mmol/l EGTA, 1 mmol/l sodium orthovanadate, 2.5 mmol/l sodium pyrophosphate, 1 mmol/l β-glycerophosphate, 1 mmol/l phenylmethyl sulfofluoride, 1 μg/ml pepstatin, aprotinin and leupeptin). After sonication, the cells were incubated on ice for 15 min and centrifuged at 14,000 rpm for 10 min at 4°C. The supernatants containing cell lysates were collected, and the protein concentrations were measured using the method of Bradford and coworkers [40].

### Western blotting

The cytoplasmic extracts (15 and 30 μg of protein was loaded for p38 and JNK western blotting, respectively) were diluted in Laemmli buffer and boiled at 95°C for 5 min. Proteins were separated by 8% SDS-PAGE and transferred onto PVDF membranes by electroblotting. The membranes were blocked for 2 hours at room temperature in 5% nonfat dry milk in Tris-buffered saline-Tween (TBS-T) and then washed three times with TBS-T. The membranes were incubated overnight at 4°C with phospho-anti-MAPK (1:1000) antibodies in 3% BSA TBS-T. After washing with TBS-T, blots were incubated with a horseradish peroxidase conjugated anti-rabbit antibody. The protein complexes were visualized by chemiluminescence using the ECL Western blotting detection reagents (Amersham Pharmacia Biotech Inc., Orsay, France). The membranes were subsequently stripped and reprobed with anti-total MAPK (1:1000 for total p38, and 1:500 for total JNK) antibodies.

### RNA isolation and RT-PCR

Nonadherent chondrocytes were treated for 60 min with pharmacological inhibitors and then stimulated with OCP crystals or IL-1β. After cell collection, total RNA was isolated using TRIzol reagent, in accordance with the manufacturer's instructions. Then, 2 μg of each sample was reverse transcribed at 37°C for 50 min using the M-MLV RT-PCR system. The resulting cDNA samples were amplified by PCR.

The PCR primers for bovine iNOS [41] were as follows: sense, 5'-TAG AGG AAC ATC TGG CCA GG-3', corresponding to positions 682–701; and antisense, 5'-TGG CAG GGT CCC CTC TGA TG-3', corresponding to positions 1034-053. These amplified a 372-bp product. The primers for IL-1β [37] were as follows: sense, 5'-TAC CTG AAC CCA TCA ACG AAA-3', corresponding to positions 517–533; and antisense, 5'-GAT GAA TGA AAG GAT GCC CTC-3', corresponding to positions 799–783. These amplified a 275-bp product. The collagen IIα1 primers [42] were as follows: sense, 5'-GAT CCG CAA CAT GGA GAC TGG CGA-3'; and antisense, 5'CAA GAA GCA GAC AGG CCC TAT GTC CAC-3'. These generated a 527-bp product. For the housekeeping gene GAPDH (glyceraldehyde-3-phosphate dehydrogenase), the sense primer was 5'-ATC ACC ATC TTC CAG GAG CG-3', corresponding to positions 245–264, and the antisense primer was 5'-CCT GCT TCA CCA CCT TCT TG-3', corresponding to positions 817-798, which amplified a 579-bp product. The PCR products were analyzed by electrophoresis on 2% agarose gel containing ethidium bromide.

### Statistical analysis

Each experiment was conducted at least three times. Data are presented as the mean ± standard deviation unless indicated otherwise. For the statistical analysis, *post hoc *tests were done when analysis of variance results were significant. *P *< 0.05 was considered statistically significant. Statistical analyses were conducted using GraphPad software (San Diego, CA, USA).

## Results

### Octacalcium phosphate crystals stimulate NO production by both articular cartilage fragments and isolated articular chondrocytes, and induce iNOS mRNA in isolated articular chondrocytes

**Figure 1:**
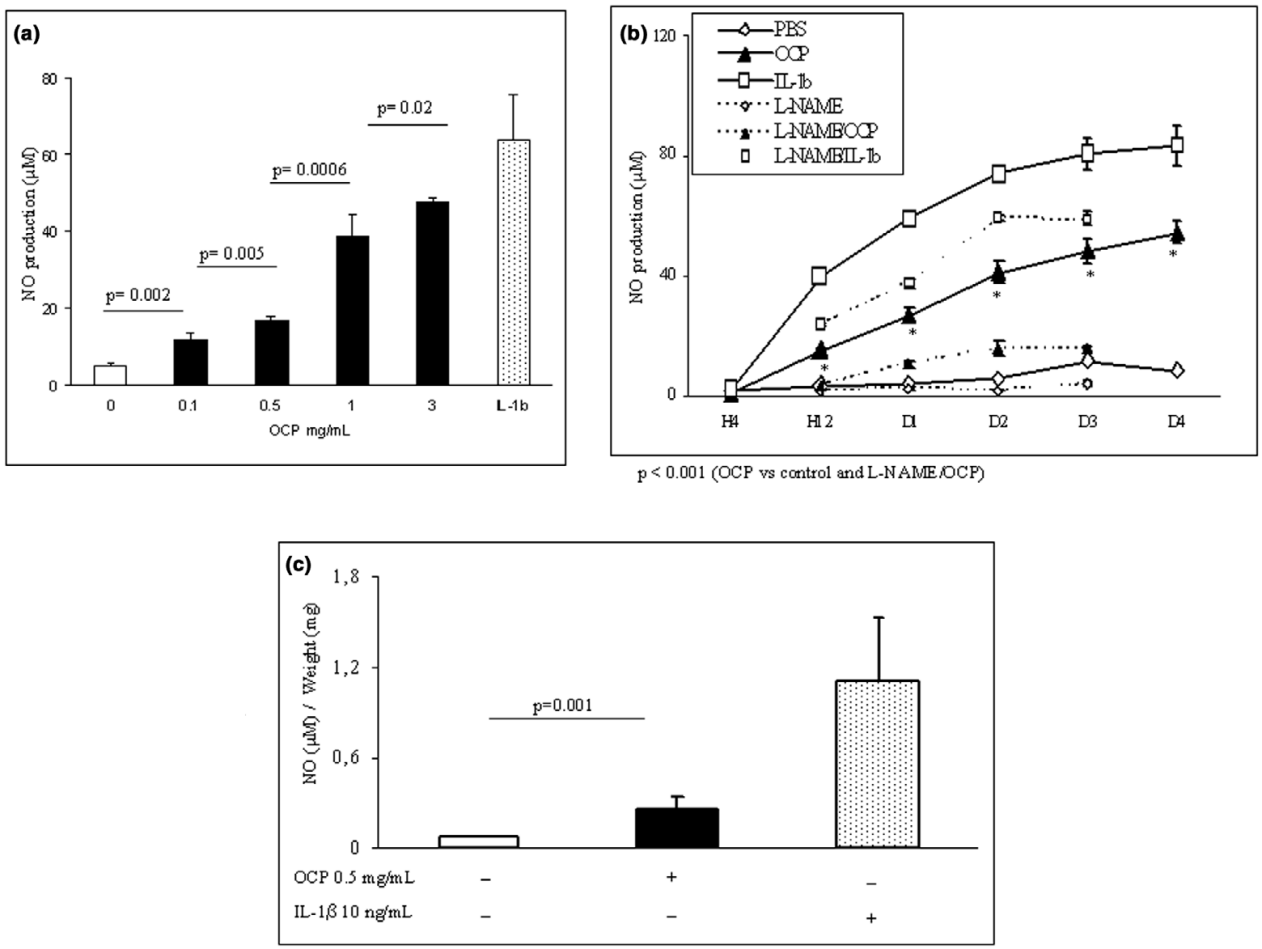
OCP crystal-induced NO release by articular cartilage fragments and isolated articular chondrocytes. Nonadherent articular bovine chondrocytes (10^6 ^cells/ml) were stimulated with OCP crystals (see Materials and method). Articular cartilage fragments were dissected and cultured as described in the Materials and method section. NO accumulation was determined with the Griess reaction. **(a) **Dose–response NO production by chondrocytes was determined 24 hours after stimulation with different doses of OCP crystals or IL-1β. **(b) **Kinetics of NO release was determined after stimulation with OCP crystals (0.5 mg/ml), IL-1β (10 ng/ml) alone or preincubated with L-NAME (2 mmol/l), a nonspecific iNOS inhibitor (*n *= 3 different experiments). **(c) **NO released by articular cartilage disks, and reported to cartilage dry weight, were measured 4 days after OCP crystals or IL-1β (10 ng/ml) stimulation (*n *= 3). *P < 0.001, OCP versus control, L-NAME/OCP). L-NAME, *N*^G^-nitro-L-arginine methyl ester; OCP, octacalcium phosphate; PBS, phosphate-buffered saline.

**Figure 2:**
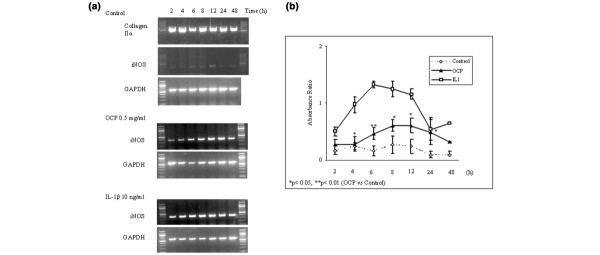
Stimulation of NO production and induction of iNOS mRNA expression by OCP crystals. Nonadherent bovine articular chondrocytes were stimulated with OCP crystals (0.5 mg/ml) or IL-1β (10 ng/ml). **(a) **iNOS and collagen II transcripts were detected in cell pellets by RT-PCR (see Materials and method). **(b) **Absorbance ratio (iNOS/GAPDH) of the RT-PCR. The RT-PCR was representative of three independent experiments, while NO release (triplicate experiments) and the absorbance ratio were expressed as mean ± standard deviation from the same three experiments. *P < 0.05, **P < 0.01, OCP versus control. GAPDH, glyceraldehyde-3-phosphate dehydrogenase; iNOS, inducible nitric oxide synthase; OCP, octacalcium phosphate.

To investigate the role of OCP crystals in cartilage destruction, we isolated bovine articular cartilage fragments and chondrocytes. Isolated articular chondrocytes were cultured in poly-HEMA-coated plates, which is a nonadherent culture condition previously shown to prevent chondrocyte de-differentiation [38]. OCP crystals induced dose-dependent NO production (Fig. 1a) by normal isolated articular chondrocytes incubated with the crystals for 24 hours. Under these conditions, we checked that the nonadherent cells consistently expressed collagen II mRNA 48 hours after stimulation (Fig. 2a). Significant NO release was achieved with crystal concentrations as low as 0.1 mg/ml, which is a level known to occur in human synovial fluids. Between 1 and 3 mg/ml, a plateau was reached, with no cytotoxic effects as assessed by trypan blue exclusion (data not shown). We therefore used 0.5 mg/ml of OCP crystals in further experiments.

Time-dependent stimulation of NO production was observed, with significant levels as early as 8 hours after stimulation and a further increase until the 4-day time point, with no plateau (Fig. 1b). L-NAME, a nonspecific iNOS inhibitor, reduced NO production by OCP crystal- and IL-1 stimulated chondrocytes (Figure 1b). With articular cartilage fragments, statistically significant NO production induced by OCP crystals was found only 4 days after stimulation (Fig. 1c), whereas NO production was detected 24 hours after IL-1β stimulation (data not shown). As previously demonstrated [43], IL-1β stimulated NO production by both isolated chondrocytes and articular cartilage organ culture, and increased iNOS mRNA expression (Fig. 1a–c). NO production was associated with time-dependent induction of iNOS mRNA expression, which was increased after 4 hours, reached a plateau between 8 and 24 hours, and decreased 48 hours after stimulation (Fig. 2a,b).

### Octacalcium phosphate crystal-induced NO production was regulated at both transcriptional and translational levels

**Figure 3:**
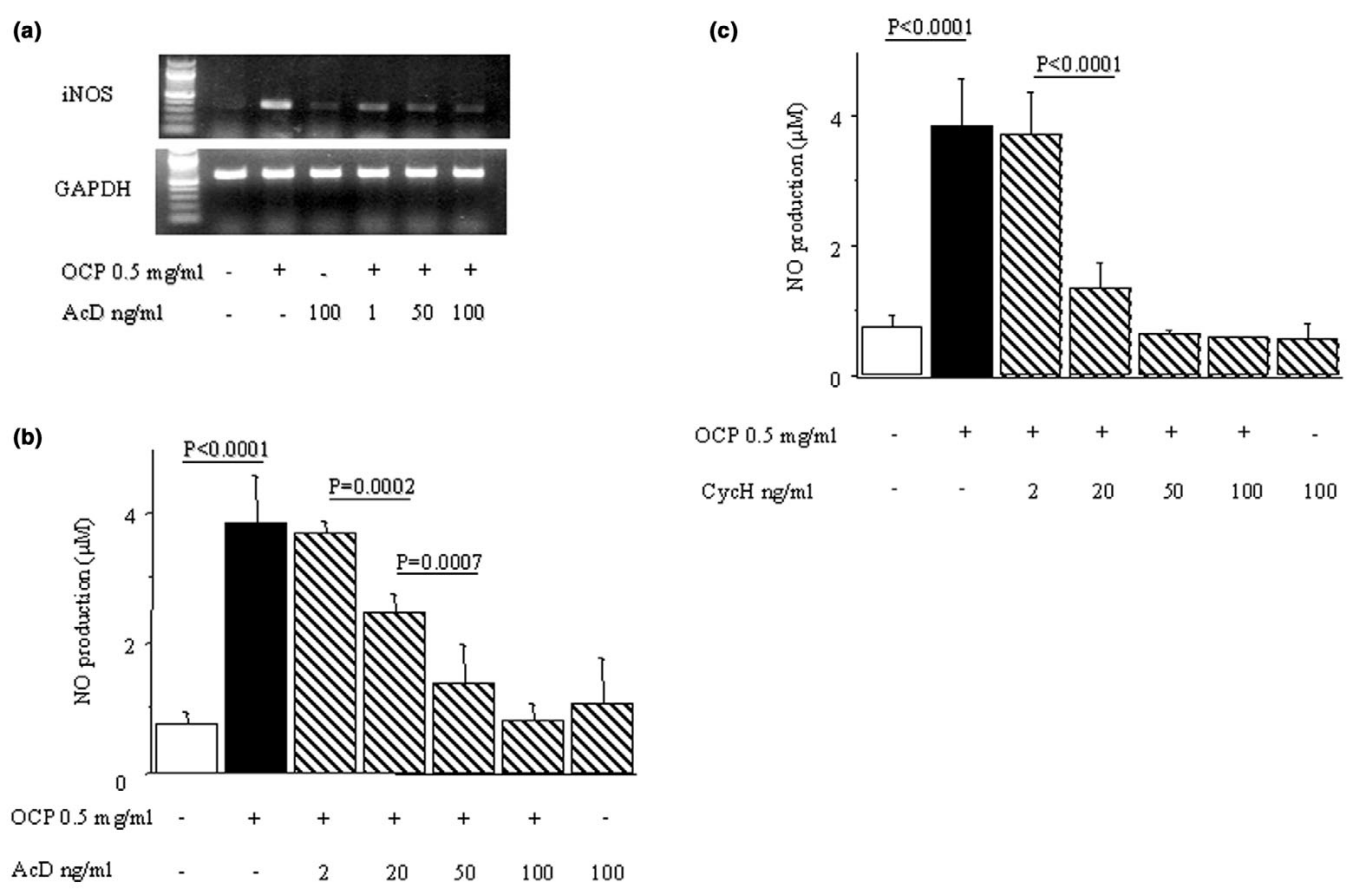
OCP crystal-induced NO production is dependent on both transcription and translation. Bovine articular chondrocytes cultured on poly-HEMA-coated plates were stimulated with OCP crystals or IL-1β for 24 hours with or without pretreatment with **(a,b) **AcD, an inhibitor of transcription, or **(c) **CycH, an inhibitor of translation. AcD, actynomycin D; CycH, cycloheximide; GAPDH, glyceraldehyde-3-phosphate dehydrogenase; NO, nitric oxide; OCP, octacalcium phosphate; poly-HEMA, poly-(2-hydroxyethyl methacrylate).

As shown in Fig. 2a, OCP crystals induced iNOS mRNA transcription in articular chondrocytes, followed 4 hours later by NO production. NO production and iNOS mRNA expression were inhibited when chondrocytes were pre-incubated for 1 hour with the transcription inhibitor actinomycin D. This effect was dose-dependent, being significant with an actinomycin D concentration as low as 20 ng/ml (Fig. 3a,b). With 100 ng/ml actinomycin D, no toxic effect was detected by trypan blue exclusion. Levels of iNOS mRNA decreased after 24 hours of stimulation, whereas NO production continued to increase for 4 days, suggesting post-transcriptional regulation. This was confirmed when preincubation of chondrocytes with the translation inhibitor cycloheximide at a dose as low as 20 ng/ml resulted in a significant decrease in OCP crystal induced NO release (Fig. 3c). Thus, NO production induced by OCP crystals was regulated at both transcriptional and post-transcriptional levels, as observed with IL-1β (data not shown).

### Octacalcium phosphate crystals induced IL-1β expression, but octacalcium phosphate crystal induced iNOS mRNA and NO production were independent of IL-1β

**Figure 4:**
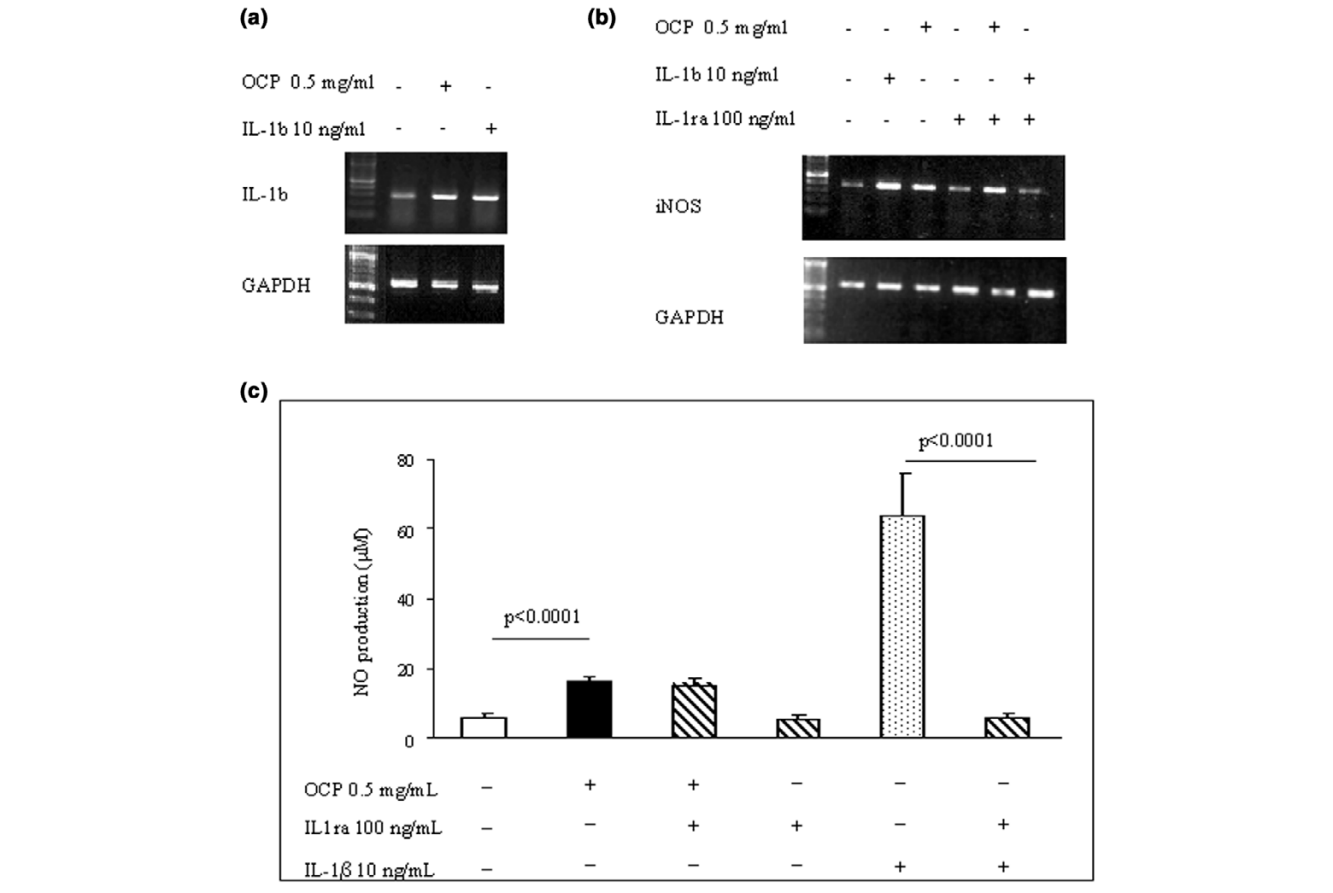
OCP crystal-induced iNOS mRNA expression and NO release is not IL-1β dependent. Nonadherent articular chondrocytes were stimulated with OCP crystals or IL-1β for 24 hours with or without pretreatment with IL-1ra for 1 hour. **(a,b) **IL-1β and iNOS transcripts were assessed by RT-PCR and **(c) **NO release was measured in the supernatants. The RT-PCR was representative of three experiments. GAPDH, glyceraldehyde-3-phosphate dehydrogenase; IL-1ra, IL-1 receptor antagonist; iNOS, inducible nitric oxide synthase; NO, nitric oxide; OCP, octacalcium phosphate.

Previous experiments have shown that BCP crystals induced the production of inflammatory cytokines, including IL-1α, IL-6, TNF-α and IL-8, by peripheral adherent monocytes (Prudhommeaux and coworkers, Champy and coworkers, unpublished data) and TNF-α by macrophages [44]. Here, we demonstrated that OCP crystals induced IL-1β mRNA expression (Fig. 4a). When we pretreated chondrocytes with IL-1 receptor antagonist for 1 hour before stimulation with either OCP crystals or IL-1β, we found that IL-1 receptor antagonist completely inhibited IL-1β-induced iNOS mRNA expression and NO production but had no influence on the effects of OCP crystals (Fig. 4b,c). This suggested that iNOS gene expression induced by OCP crystals was not mediated by a paracrine or autocrine mechanism involving the IL-1β receptor.

### p38 and JNK MAPKs are activated by octacalcium phosphate crystals and are involved in octacalcium phosphate crystal induced iNOS expression and NO production

**Figure 5:**
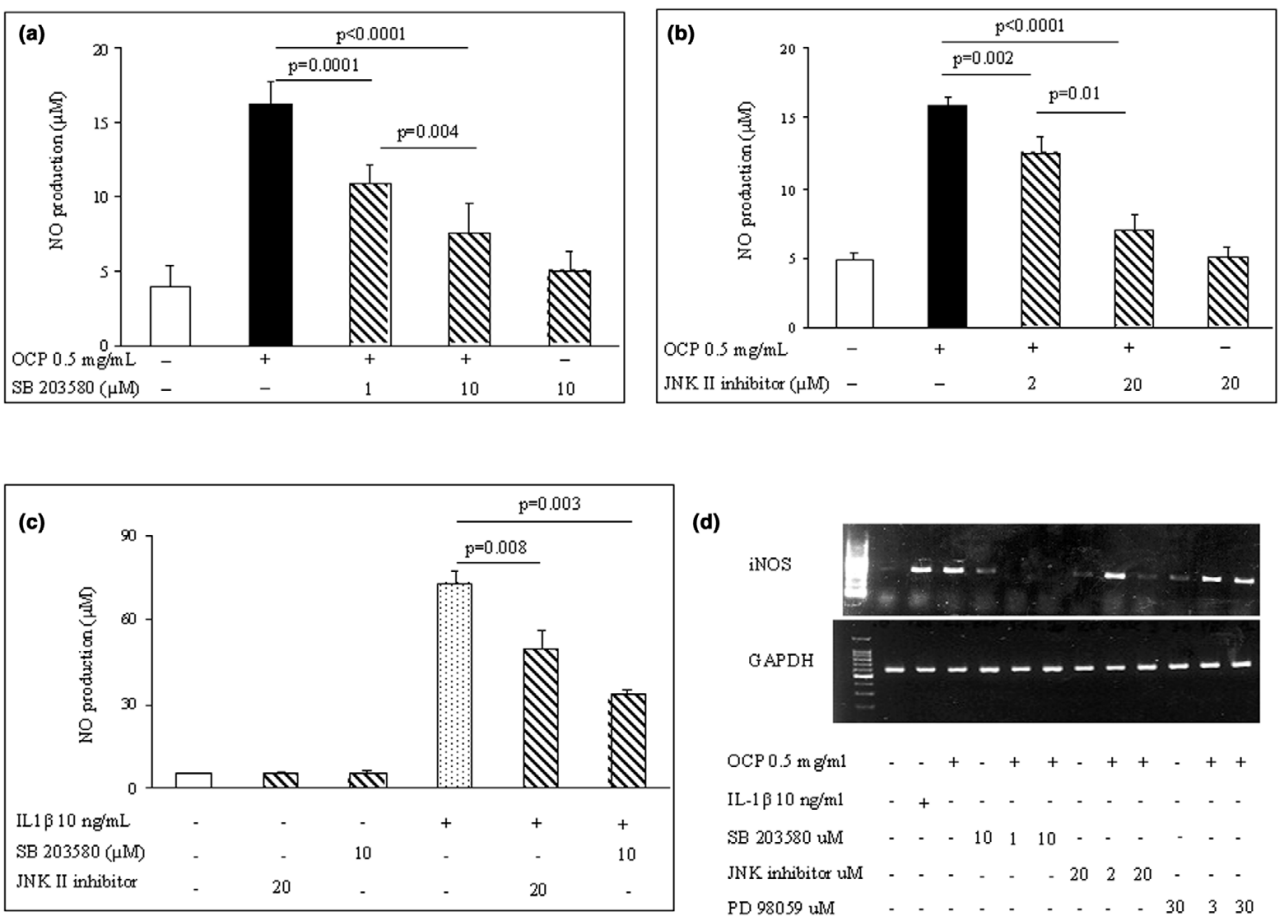
p38 and JNK MAPK pathways regulate OCP crystal induced NO production and iNOS mRNA expression. Nonadherent bovine articular chondrocytes were stimulated with OCP crystals or IL-1β for 24 hours with or without pretreatment (1 hour) with **(a,c) **SB 203580 (a p38 MAPK inhibitor), **(b,c) **JNK II inhibitor or **(d) **PD 58059 (an Erk1/2 MAPK inhibitor). iNOS transcripts (panel d) were assessed 8 hours after stimulation by OCP crystals or IL-1β by RT-PCR. The RT-PCR was representative of three experiments. GAPDH, glyceraldehyde-3-phosphate dehydrogenase; iNOS, inducible nitric oxide synthase; JNK, c-Jun amino-terminal kinase; NO, nitric oxide; OCP, octacalcium phosphate.

**Figure 6:**
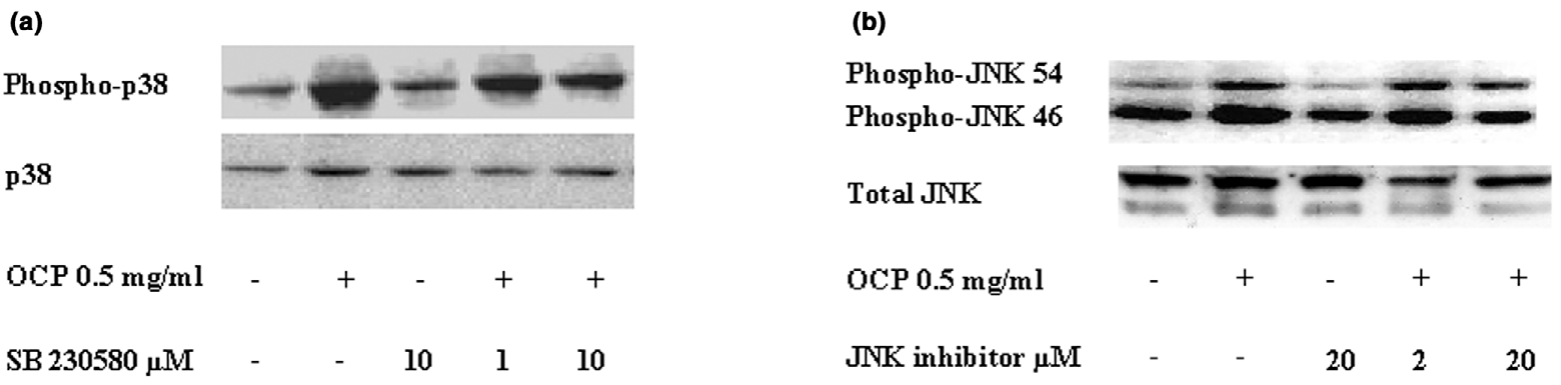
OCP crystal induced p38 and JNK MAPK activation. Nonadherent bovine articular chondrocytes were stimulated with OCP crystals for 20 min with or without pretreatment (1 hour) with SB 203580 or JNK II inhibitor. Fifteen (p38 MAPK) or 30 μg (JNK MAPK) protein of cell lysates were subjected to SDS-PAGE/Western blot analysis. The blots were probed with phosphospecific **(a) **p38 and **(b) **JNK MAPK antibodies. To ensure equal loading, the blot was stripped and reprobed with total p38 MAPK or JNK antibodies. JNK, c-Jun amino-terminal kinase; OCP, octacalcium phosphate.

Because most studies support a role for MAPK pathways in the regulation of iNOS expression, we used pharmacological inhibitors to investigate the potential role for these pathways in OCP crystal-induced iNOS gene expression and NO production. The p38 MAPK inhibitor SB 203580, at a concentration as low as 1 μmol/l significantly reduced NO production (Fig. 5a) and completely inhibited iNOS mRNA induction by crystals (Fig. 5d). JNK II inhibitor also reduced NO production, at a concentration as low as 2 μmol/l (Fig. 5b). Conversely the p42/44 MAPK inhibitor PD 98059 had no effect, even at a high concentration (30 μmol/l, data not shown). Results were similar with IL-1β used as a positive control (Fig. 5c). These findings support involvement of both the p38 and the JNK MAPK pathways in OCP crystal-induced NO production. We then used immunoblotting to show that OCP crystals induced p38 and JNK phosphorylation and that this effect was inhibited by chondrocyte pretreatment with p38 or JNK MAPK inhibitors, respectively (Fig. 6a,b).

**Figure 7:**
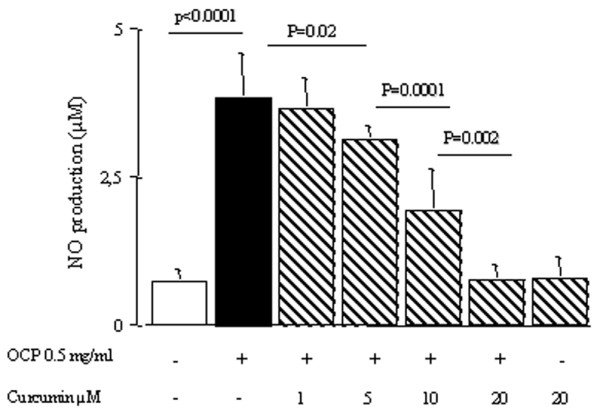
Role of AP-1 transcription factor in the induction of NO production by OCP crystals. Nonadherent bovine articular chondrocytes were stimulated with OCP crystals or IL-1β for 24 hours with or without pretreatment (1 hour) with various doses of curcumin, an AP-1 inhibitor. NO release was measured in the supernatants. AP = activator protein; NO = nitric oxide; OCP = octacalcium phosphate;

Because BCP crystals activated c-Jun and c-Fos [45], and the transcriptional factor activator protein (AP)-1 has been implicated in iNOS regulation, we investigated the role for AP-1 in OCP crystal-induced NO production. NO production decreased significantly when chondrocytes were preincubated with curcumin, an AP-1 inhibitor directed toward heterodimerization of c-jun-c-fos, at a concentration as low as 1 μmol/l. This inhibition of NO production was dose dependent (Fig. 7).

## Discussion

OCP crystals, the most potent inflammation-inducing BCP crystals [33,34], are found in joint fluids [35]. In our study, OCP crystals induced iNOS gene expression by isolated articular chondrocytes and NO production by both isolated chondrocytes and cartilage fragments. The expression of iNOS mRNA increased 4 hours after BCP crystal stimulation, peaked between 8 and 12 hours, and decreased after 24 hours, in agreement with findings reported by Terkeltaub and coworkers [37] and by Adler and colleagues [41] after stimulation by monosodium urate (MSU) crystals and *Salmonella dublin*, respectively. NO production was significant in culture supernatants as early as hour 8 and increased throughout the 4-day poststimulation period. Using a nonspecific iNOS inhibitor, L-NAME, we observed that the inhibition of NO production was less marked after IL-1 stimulation compared with OCP crystals. This could result from the magnitude of the effect achieved by each reagent.

Previous studies suggested that BCP crystals may directly activate articular chondrocytes to produce MMP-13 [16], collagenase [12] and other MMPs capable of degrading cartilage, as well as to release prostaglandin [13]. Although chondrocytes can ingest BCP crystals [13], BCP-induced MMP production does not require intracellular dissolution of the crystals [46]. In fibroblasts 4 hours after stimulation by BCP crystals, BCP crystal endocytosis was not yet complete and solubilization of the crystals had not yet occurred [47]. In addition, BCP crystal phagocytosis by chondrocytes has been observed after 24 hours [13], suggesting that in the present study OCP crystal-induced iNOS expression did not require this process.

The direct effect of OCP crystals on cells may involve an interaction between OCP crystals and cell surface receptor-like structures, such as integrins [37]. Proteins bind to BCP crystals, including OCP, and to MSU crystal surfaces [34,48,49] and can modulate cell function *in vivo *and *in vitro*. In addition, fibronectin fragments induced p38 and JNK MAPK activation via interactions with integrins [50]. Tan and coworkers [51] reported regulation of iNOS expression by an integrin-linked kinase, which was an ankyrin repeat containing a serine/threonine protein kinase that interacted with the cytoplasmic domain of β_1 _integrin [51]. Furthermore, Liu and Lioté [37] found that, within a few minutes, MSU crystals activated several molecules linked to the focal adhesion kinase complex, most notably Pyk-2, which appeared to be central to p38 MAPK activation and promoted iNOS gene expression, NO production and MMP-3 translation.

NO regulates chondrocyte and cartilage functions and often acts as an IL-1β second messenger. Although *in vitro *NO had either catabolic [52-57] or anabolic [58-61] effects, *in vivo *studies clearly demonstrated that inhibition of NO production by *N*-iminoethyl-L-lysine reduced the progression of structural damage in rabbits with experimentally induced OA [30,32]. NO inhibition was associated with reductions in MMP synthesis, IL-1β and prostaglandin E_2 _production, and chondrocyte apoptosis [32]. As it has been demonstrated that IL-1β-induced MMP production is NO dependent [54], the mechanism of BCP crystal induced MMP-13 production by chondrocytes remains unknown [16]. One possibility is a direct effect, similar to the induction of MMP-1 production by fibroblasts stimulated by BCP crystals [20]. Alternatively, NO mediation may be involved, as shown for MSU crystal-induced MMP-3 in chondrocytes [37].

Although this is the first report on iNOS production by bovine articular chondrocytes treated with BCP crystals, iNOS expression has been consistently described in chondrocytes treated with various inflammatory cytokines, including interferon-γ, TNF-α and IL-1β [43,62,63]. IL-1β seems to be the key cytokine in the induction of cartilage catabolism and plays a pivotal role in the cartilage destruction typical of OA [64]. In our experiments using IL-1 receptor antagonist, we found that, although OCP crystals induced IL-1β mRNA, iNOS mRNA expression induced by OCP crystals were in part independent from IL-1β. In contrast, iNOS mRNA expression and NO production induced by IL-1β were inhibited by IL-1 receptor antagonist. These results are in agreement with MSU crystal induced iNOS expression, as observed by Liu and Lioté [37].

As described for MSU crystals, IL-1β and OCP crystal induced iNOS mRNA expression by chondrocytes required the p38 and JNK MAPK pathways, whereas Erk1/2 MAPK was not involved. NO production was partially inhibited by low p38 inhibitor concentration whereas iNOS mRNA expression was markedly decreased, reflecting differences between iNOS protein production and activity and transcriptional regulation. Furthermore, NO production was assessed after 24 hours of stimulation and iNOS mRNA expression at 8 hours. A preferential role for p38 and JNK MAPK in iNOS activation was observed by Mendes and coworkers using IL-1β stimulation [65]. However, depending on the stimulus and the cell type, MAPK may play a positive, negative, or neutral role in regulating iNOS expression. For instance both the p38 and p42/44 MAPK pathways have been reported to be involved in iNOS induction by IL-1β-stimulated cardiomyocytes [66]. Conversely, in pulmonary vascular smooth muscle cells, p38 MAKP inhibited IL-1β-mediated iNOS expression [67]. Also, BCP crystal induced MMP-1 activation in skin fibroblasts was activated by p42/44 MAPK [20]. Two hypotheses may explain the variations in the role of p38 MAPK on iNOS expression. One is that p38 MAPK exists as several isoforms, each expressed by specific cell types; under this hypothesis, the p38 MAPK inhibitors used in previous work and in the present study may not be sufficiently selective to inhibit one specific p38 MAPK isoform. In support of this possibility, Guan and coworkers [68] demonstrated that p38α MAPK isoform activation was required for IL-1β-induced stimulation of iNOS production by rat glomerular mesangial cells; thus, overexpression of the kinase inactive mutant form of p38α MAPK inhibited IL-1β-induced iNOS expression. The second hypothesis is that regulation of iNOS promoter may be complex and specific for each cell type [69].

Structural analysis of the 5' flanking region of the iNOS gene has identified multiple binding sites for the transcriptional factor AP-1 [70], a heterodimer that is composed of the protein products of Fos and Jun. BCP crystals induce c-*jun, c-fos *and AP-1 in human fibroblasts [20,46]. In this study JNK MAPK was also activated by OCP crystals, and OCP crystal induced NO production was inhibited by curcumin, a c-fos/c-jun heterodimerization inhibitor. These results suggest a role for AP-1 in OCP crystal induced iNOS gene expression, as demonstrated by Marks-Konczalik and coworkers [70] in A549 cells (human alveolar type II lung carcinoma cell line) stimulated by a mixture of cytokines containing interferon-γ, TNF-α and IL-1β. Inhibition of iNOS by actinomycin and cycloheximide may reflect, at least in part, inhibition of AP-1 complex synthesis, normally induced by BCP crystals.

## Conclusion

In summary, this study showed that OCP crystals, a member of the family of BCP crystals, caused inflammation by directly activating chondrocytes to induce IL-1β and iNOS gene expression and NO production. OCP crystal induced iNOS activation was IL-1β independent, and involved the p38 and JNK MAPK pathways, probably under AP-1 control. These results demonstrated that chondrocytes may play a direct and active role in cartilage destruction by specific microcrystals.

## Abbreviations

AP = activator protein; BCP = basic calcium phosphate; bp = base pair; CPPD = calcium pyrophosphate dihydrate; DMEM = Dulbecco's modified Eagle's medium; FBS = foetal bovine serum; IL = interleukin; JNK = c-Jun amino-terminal kinase; L-NAME = *N*^G^-nitro-L-arginine methyl ester; OA = osteoarthritis; OCP = octacalcium phosphate; MAPK = mitogen-activated protein kinase; MMP = matrix metalloproteinase; MSU = monosodium urate; NO = nitric oxide; NOS = nitric oxide synthase; poly-HEMA = poly-(2-hydroxyethyl methacrylate); RT-PCR = reverse trasncription polymerase chain reaction; TBS-T = Tris-buffered saline-Tween; TNF = tumour necrosis factor.

## Competing interests

The author(s) declare that they have no competing interests.

## Authors' contributions

HKE participated in study design, contributed to all experiments, analyzed results, and prepared and wrote the manuscript. BU contributed to PCR studies and chondrocyte cultures. CR prepared and analyzed OCP crystals. FL contributed to study design, analyzed results, prepared, wrote the manuscript and supervised the work.
